# Mapping Water Stress Incidence and Intensity, Optimal Plant Populations, and Cultivar Duration for African Groundnut Productivity Enhancement

**DOI:** 10.3389/fpls.2017.00432

**Published:** 2017-03-29

**Authors:** Vincent Vadez, Oumarou Halilou, Halime M. Hissene, Pierre Sibiry-Traore, Thomas R. Sinclair, Afshin Soltani

**Affiliations:** ^1^Crop Physiology Laboratory, International Crops Research Institute for the Semi-Arid TropicsPatancheru, India; ^2^Sahelian Center, International Crops Research Institute for the Semi-Arid TropicsNiamey, Niger; ^3^Department of Biology, Faculty of Sciences and Techniques, Abdou Moumouni UniversityNiamey, Niger; ^4^Centre d’Etude Régional pour l’Amélioration de l’Adaptation à la SécheresseThiès-Escale, Sénégal; ^5^Samanko Station, International Crops Research Institute for the Semi-Arid TropicsBamako, Mali; ^6^Department of Crop Sciences, North Carolina State University, RaleighNC, USA; ^7^Agronomy Group, Gorgan University of Agricultural Sciences and Natural ResourcesGorgan, Iran

**Keywords:** agronomy, sowing density, risk assessment, crop modeling, livelihood

## Abstract

Groundnut production is limited in Sub-Saharan Africa and water deficit or “drought,” is often considered as the main yield-limiting factor. However, no comprehensive study has assessed the extent and intensity of “drought”-related yield decreases, nor has it explored avenues to enhance productivity. Hence, crop simulation modeling with SSM (Simple Simulation Modeling) was used to address these issues. To palliate the lack of reliable weather data as input to the model, the validity of weather data generated by Marksim, a weather generator, was tested. Marksim provided good weather representation across a large gradient of rainfall, representative of the region, and although rainfall generated by Marksim was above observations, run-off from Marksim data was also higher, and consequently simulations using observed or Marksim weather agreed closely across this gradient of weather conditions (root mean square of error = 99 g m^-2^; *R*^2^ = 0.81 for pod yield). More importantly, simulation of yield changes upon agronomic or genetic alterations in the model were equally predicted with Marksim weather. A 1° × 1° grid of weather data was generated. “Drought”-related yield reduction were limited to latitudes above 12–13° North in West Central Africa (WCA) and to the Eastern fringes of Tanzania and Mozambique in East South Africa (ESA). Simulation and experimental trials also showed that doubling the sowing density of Spanish cultivars from 20 to 40 plants m^-2^ would increase yield dramatically in both WCA and ESA. However, increasing density would require growers to invest in more seeds and likely additional labor. If these trade-offs cannot be alleviated, genetic improvement would then need to re-focus on a plant type that is adapted to the current low sowing density, like a runner rather than a bush plant type, which currently receives most of the genetic attention. Genetic improvement targeting “drought” adaptation should also be restricted to areas where water is indeed an issue, i.e., above 12–13°N latitude in WCA and the Eastern fringes of Tanzania and Mozambique.

## Introduction

Groundnut is cultivated in environments where it seems the crop may experience water deficits, but there is no thorough geo-referenced assessment of where such water deficits are a problem for peanut production. It is indeed often considered that water limitation (or so called “drought”) is one of the main issues limiting crop yield, especially in Sub-Saharan Africa (SSA) but also in South Asia. However, peanut transpiration water needs to grow a fully irrigated crop in a regular rainy season was about 30 L plant^-1^, which could be extrapolated to 450 mm at the sowing density used in these trials (Halilou et al., 2015). Assuming an equivalent amount of water evaporates from the soil during a cropping season, a peanut crop would be fully irrigated with 900 mm, of course provided the rains are equally distributed during the season. Yet, much of the environments where groundnut is cultivated in SSA receive at least 900 mm. Hence, where and how much groundnut productivity is limited by water availability is an important question to address in the scope of a priority setting of breeding targets. This study then proposes to test to what extent and where water is a substantial limitation for growing peanut. We hypothesized that this restriction might only prevail in the driest part of the semi-arid tropics, especially in the North Sahelian zone.

In a recent paper (Vadez et al., 2016), the robustness of the Simple Simulation Modeling (SSM) model to predict peanut yield was demonstrated, where the model accurately simulated yield across water regimes leading to a wide range of yields (170–480 g m^-2^). To that end 16 trials were carried out between 2008 and 2014 in India and Niger, under either fully irrigated or intermittent water stress conditions (reported in Hamidou et al., 2012, 2013). The model was set to simulate a representative Spanish cultivar and the root mean square of error (RMSE) of the relationship between the observed and the simulated pod yield was 16% of the average observed pod yield, indicating robustness of the model to simulate pod yield across a range of water regimes and across varied environments (Vadez et al., 2016). This model is similar in many ways to a previously developed groundnut model and also successfully tested in Australia (Hammer et al., 1995). A validation of the model’s simulation of the leaf canopy development has been recently reported (Halilou et al., 2016). The CSM-CROPGRO-peanut model (Narh et al., 2015) has also been used for groundnut in SSA and resulting in a RMSE of 26% of the average yield after using multiple steps of calibration of the model coefficients in a site-specific manner. The decision to use the SSM model was in its simplicity (see Soltani and Sinclair, 2015 for a comparison to other models) and in its independence on calibration steps like in the CSM-CROPGRO-peanut, Cropping System Model Crop Growth. The SSM model requires daily radiation, minimum/maximum temperature, and rainfall data.

A successful spatial analysis of modeling outputs usually requires at least 20 years of weather data. While such extensive data are usually available in developed countries, they are severely lacking in many developing countries and weather generators have been created to palliate that gap. Weather generators basically take into account the mean climate variables, and then generate data based on an analysis of variability across years and among days within years. A key question is whether such data can be used in crop simulations to reliably assess crop productivity across locations. Once it was established that the generated weather data was adequate, the first task to which the model was applied was to identify those regions which are found to exhibit yield limitations due to water-deficits at some point during the growing season. Here, it should be noted that prediction were made on historical data and attempts to address possible changes in future climate and related productivity output, although very important, were out of the scope of this study.

Next, it was hypothesized that groundnut yield could be limited in short duration types, and that the current sowing density of groundnut in SSA could also be a major yield-limiting factor. In Africa, breeders indeed often seek to breed for short-duration cultivars, which makes sense in certain crops that have a ceiling of water availability (e.g., post-rainy sorghum or chickpea in the semi-arid tropics of India). By contrast, for crops that receive rainfall during the entire growing season short duration means also a shorter time for light interception and a potential yield penalty. This view is supported by recent study showing the advantage of cultivar types with a longer life cycle (Narh et al., 2014). The management alteration of plant density may also be important because it has a direct influence on the amount of water available to each plant, and also to the profile of light interception by the crop canopy. This topic has received limited attention (Giayetto et al., 1998; Tewolde et al., 2002). Peanut cultivars can be of runner types (e.g., Virginia runner types) or bush types (e.g., Spanish bunch types), each type leading to much different leaf architecture. A study in four Virginia type cultivars showed that an increased plant density leads to increased vegetative development and more numerous reproductive organs, although this did not lead to higher yield because of indeterminacy in pod setting (Cahaner and Ashri, 1974). A study in two Virginia type cultivars showed no increase in the vegetative growth and yield at higher density (Tewolde et al., 2002). By contrast, Spanish cultivar were shown to be responsive to increased densities (Babu et al., 1984) and in India, where Spanish types are predominantly grown, recommended densities are well above 30 plant m^-2^. In Africa, runner types were predominantly cultivated earlier. The demand for shorter duration types has favored bush types and these are now predominantly cultivated. However, during that transition the agronomic management of the crop has remained the same and the recommended seed rates are still typically 60 kg ha^-1^, which amounts to about 20 plants m^-2^ (assuming a seed size of 30 g/100 seeds). Therefore, two specific potential alterations for peanut production were explored with the model: (i) plant genetic alteration to optimize the growing season duration of the crop, and (ii) management alteration to optimize plant density to maximize crop yield.

Therefore, the objectives of this work were to assess: (i) the relevance of modeling outputs generated from Marksim weather as compared to observed weather from Mali; (ii) the extent of the water limitation of yield across two main production blocks of SSA, (iii) the optimal planting density for maximizing yield, (iv) the effect of breeding shorter duration cultivars.

## Materials and Methods

### Model Structure

The SSM-legume model of Soltani and Sinclair (2011) was used in this study. The SSM model has proven to be robust under a wide range of environments and a number of crop species, for instance in chickpea (Soltani and Sinclair, 2011; Vadez et al., 2012, 2013), lentil (Ghanem et al., 2015a,b), bean (Marrou et al., 2014), soybean (Sinclair et al., 2014), and peanut (Vadez et al., 2016). The model simulates phenological development on the basis of biological day accumulation (Soltani and Sinclair, 2011) to reach the different phenological stages. The concept of biological days is based primarily on optimal temperature, but also assumes sufficient water and nutrients as critical variables. The general structure of the model is to simulate phenological development on the basis of cumulative temperature units to reach the different phenological stages. The model simulates leaf development by means of a phyllochron that expresses the temperature units needed for a new leaf to appear on the main stem and a power relationship between the number of leaves on the main stem and plant leaf area. The resulting leaf area index is used to simulate light intercepted and then calculate the mass accumulated each day from a radiation use efficiency coefficient. The model partitions accumulated mass between the plant organs, and simulates a plant nitrogen balance. The model simulates yield formation during the reproductive phase using a coefficient that reflects a daily increase in the harvest index. Soil water stress is calculated daily by the model based on the water required to support mass accumulation and the available soil water. Leaf expansion, mass accumulation, and symbiotic nitrogen fixation are all retarded if the amount of available soil water has decreased below each threshold where activity is limited.

#### Plant Development

In the SSM model, phenological development stages are defined in terms of biological days needed to reach each of these stages, namely: sowing to emergence, flowering (R1), beginning podding (R3), beginning of pod filling (R5), end of pod filling (R7), and maturity (R8). Because the daily mean temperature is often less than the optimum temperature needed for optimal plant development, the number of calendar days to achieve certain phenological stages is usually greater than the number of biological days.

The cardinal temperatures for phenological development of groundnut were set at 11°C for base temperature, 28°C for lower optimum temperature, 30°C for upper optimum temperature and 50°C for ceiling temperature (Ong, 1986; Boote et al., 1989). While the model takes into account a photoperiod effect on many of the legume species it simulates, no photoperiod effect was taken into account in the case of groundnut.

Leaf area development during the vegetative phase was based on calculation of phyllochron, which is the temperature unit duration between the appearance of two consecutive leaves on the main stem (Ong, 1986; Craufurd et al., 1997), and a key input parameter to the SSM model. A recent study by Halilou et al. (2016) on 20 groundnut genotypes confirmed that the phyllochron was constant at 56°C and similar to previous reports (Ong, 1986; Craufurd et al., 1997). A power function then relates the number of leaves on the main stem to the plant’s leaf area (Soltani and Sinclair, 2012). The study of Halilou et al. (2016) showed the mean value across genotypes of the power coefficient relating node number on the main stem and leaf area was 2.71. While there was some variation in the power coefficient among genotypes, there were only variations as a result of sowing density.

#### Crop Transpiration

Transpiration rate in the SSM model is based on the fundamental relationship between plant mass accumulation and transpiration rate (Tanner and Sinclair, 1983). Crop transpiration is calculated from the daily mass accumulation multiplied by a weighted atmospheric vapor pressure deficit (VPD) and divided by a transpiration efficiency constant of 4.5 Pa for groundnut.

To track the soil water uptake, the model calculates the current depth of soil water extraction, which is done by a simple daily increase. The daily increase in the water extraction front is simulated in the SSM model. For these simulations with peanut a value of 35 mm per biological day was assumed as daily increase in the depth of water extraction. Therefore, this parameter results in a direct estimation of the total volume of soil accessible by roots each day. The model fixes a maximum depth up to which water can be extracted, which is based on the knowledge of the soil conditions for the location where the simulations are being done.

A soil water balance is updated in the simulations each day by taking into account water addition by rainfall, irrigation, and possible increases in depth of extraction into wet soil. Soil water uptake due to crop transpiration and evaporation from the soil surface are also taken into account. The soil water balance gives a daily update of the total amount of transpirable soil water in the soil, i.e., the water available to support transpiration. Since the responses of plant processes are simulated based on the fraction of transpirable soil water (FTSW), which represents the percentage remaining of the soil water that can support plant transpiration, a daily estimate of FTSW for the soil is generated. Threshold FTSW values are then used to trigger possible limitation on mass accumulation, symbiotic nitrogen fixation, and canopy expansion.

### Comparison of Model Predictions from Observed and Marksim-Generated Weather Data

A major limitation in attempting simulations across a wide geographical area is assembling a sufficient weather data base to generate meaningful results. This is particularly true for SSA countries where collection of weather data over the past decades has been scant and heterogeneous across regions and countries. However, progress in climate science and computation has now made it possible to generate fairly accurate weather data. Here we have used the Marksim weather generator for tropical latitudes (Jones and Thornton, 2000), which is specifically developed for tropical and subtropical regions and has shown robustness over a number of studies (Jones and Thornton, 2013, 2015 and references therein). Marksim used historical weather database to run interpolation between cardinal points to generate weather data across years to reflect both the mean and the possible variation in the weather variables at a specific point. Marksim was used to generate weather files at each 1° latitude and longitude grid location. For the two large simulated areas of West (242 locations) and East Africa (246 locations), the grid locations were at approximately 100 km distances. For each location, 50 years of weather data were generated.

A subset of the Marksim-generated weather was compared to observed weather in twelve locations in Mali ranging from about 11–16° latitude. These locations in Mali allowed a test of the major gradient for weather in Mali would be North South, and in particular a large gradient in rainfall (**Table 1**). Generated weather was used to simulate groundnut yield. Our main criteria in the comparison of Marksim and observed weather was not in obtaining simulating yield that would fall exactly on a 1:1 line, but rather to test if the yield changes would be sensitive to changes of either type of weather data, i.e., if an increase/decrease in yield caused by changes in rainfall could equally be detected using Marksim or observed weather. Therefore, our main objective in this comparison was to test the closeness of the correlation between yield simulated from observed and Marksim weather. After finding a close relationship (see results below), the entire set of Marksim weather data was generated for two large blocks of locations and used to run simulation of SSM across 242 and 246 locations in West and South East Africa, respectively, for 50 years at each location. Given the wide geographical spread of the simulations results also included a number of locations where groundnut is not currently grown.

**Table 1:** Geographical coordinates and number of years in each of the locations from Mali that were used to compare Marksim-generated weather to the observed weather obtained from historical records.

	Latitude	Longitude	Number of years
Sikasso	11.35	–5.68	60
Segou	13.4	–6.15	58
Ntarla	12.7	–5.75	16
Nioro	15.23	–9.60	30
Nara	15.17	–7.28	54
Mopti	14.52	–4.10	56
Koutiala	12.4	–5.47	58
Keniebe	12.8	–11.35	47
Kayes	14.43	–11.43	54
Gao	16.27	0.05	30
Bougouni	11.42	–7.50	58
Bafoulabe	13.8	–10.83	9

### Traits Tested

#### Baseline Rainfed Yield and Yield Loss Due to Water Stress

Weather data from the West and East Africa blocks were used as input to the SSM model to simulate the grain yield under rainfed conditions. A standard sowing density of 20 plants m^-2^ was used in the simulations, which represents closely the current recommended practices for groundnut cultivation in SSA (60 kg seed ha^-1^). The model was let to decide on the sowing data, using a 60 days window starting at a time when rains are likely to start in each of the regional blocks, i.e., 20 May for West Africa and 27^th^ October in South East Africa. The criteria for sowing was when a minimum of 30 mm water in the total soil profile and 15 mm in the top soil layer (20 cm deep) had accumulated. It was also assumed that there was little or no transpirable water in the soil profile at the time the model was set to search for the sowing date. The parameters required to define the crop were those of a standard Spanish cultivar.

To assess the yield losses due to water-deficit stress, another set of simulations were run under the same agronomic conditions and weather input but in this case the model was set to irrigate the simulated crop whenever the soil dried to stress levels, therefore providing yield predictions of a fully irrigated crop. The model used a FTSW threshold for a decrease in transpiration of 0.35, which means that when the soil water balance detected a FTSW value that was equal or below 0.35, an irrigation of 40 mm was applied to the crop. The yield losses due to water-deficit stress for each pixel were then computed as the difference between the yield outputs under fully irrigated conditions from which the yield outputs under rainfed conditions were subtracted.

#### Density

Over the past decades the cultivation of Spanish type with a bushy phenotype has increased in SSA with a concomitant decrease in the proportion of Virginia runner type. However, agronomic recommendations have seemingly not been modified so the new lines are generally still sown with a plant density of 10–20 plants m^-2^. The recommendation for groundnut production in India using predominantly Spanish cultivars are for a density of 30–40 plants m^-2^. For the simulations, two planting densities were simulated, either 30 or 40 plants m^-2^, and only the data for 40 plants m^-2^ are presented (as these led to higher productivity outputs than the 30 plants m^-2^).

While increasing density increased crop productivity in most situations (see below), it may also have put peanut cultivation under higher risk in limited-rainfall conditions. Therefore, the potential yield losses due to water limitation were re-evaluated in the scope of having increased the sowing density to 40 plants m^-2^. For that purpose, simulations were also done for a fully irrigated crops with a density of 40 plants m^-2^. Yield losses caused by water deficit were then computed as the mean of modeling outputs from 50 years of weather data under rainfed conditions, subtracted from similar outputs under fully irrigated conditions.

#### Duration

Developing short duration cultivars has been a major focus of groundnut breeding programs in the past decades. However, while earliness would provide yield resilience in low rainfall environments, there is the possibility of loss in yield potential in situations of higher rainfalls. Indeed, a recent report shows the advantage of longer life cycle cultivars over short duration ones in Ghana (Narh et al., 2014). The hypothesis was then that earliness might limit crop productivity, especially in higher rainfall environments. In a number of grain legumes, earliness is defined by flowering time. In the case of groundnut, most cultivars flower quite early, i.e., in about 16–20 biological days (16 days for a standard Spanish cultivar) after sowing and the major factor affecting crop duration was the interval of time for the grain filling period (55 biological days for a standard Spanish cultivar). This period was reduced by 10 biological days to simulate a short duration cultivar. These simulations were performed using the optimal sowing density from the earlier section, i.e., 40 plant m^-2^.

### Possible Caveats

In this study covering two large blocks of West Central Africa (WCA) and East Southern Africa, we did not differentiate soil types. Soils do somewhat differ in the water holding capacity, and while this could have had important effects on a crop fed mostly on stored soil moisture, the effect of differences in soil water holding capacity would have been much less in the condition of a rainfed crop like groundnut here (i.e., receiving current rainfall rather than stored moisture). The purpose of the study was also to have an overall representation of areas where water stress is an issue and of boundary lines where water stress becomes important, and this would have been mostly driven by weather conditions (rainfall but also VPD). Finer details of these boundary lines could be obtained by narrowing down on sub-regions/specific countries.

### Experimental Tests

Two field experiments were undertaken that allowed the testing of the robustness of the simulation results. These trials are described in another paper (Halilou et al., 2016). In short, one trial was carried out in Niger and one in India. In Niger, four genotypes—two Spanish (FLEUR11 and ICG1834) and two Virginia (ICG13723 and ICG2777) genotypes—were sown in three replicated plots. Four densities (10, 20, 30, and 40 plant m^-2^) were tested in a randomized complete block design. In the lowest densities (10 and 20 plant m^-2^), each plot (4 m^2^) was made of two rows (4-m long, 50-cm distance between rows), with either 20 or 10 cm between plants. In densities 30 and 40 plant m^-2^, each plot (4 m^2^) was made of four rows (4 m long, 25 cm distance between rows), with either 14–15 or 10 cm between plants. The trial was sown during the rainy season in 2013 and was kept fully irrigated. In India, four genotypes—two Spanish genotypes (Fleur11 and ICG1834) and two Virginia genotypes (ICG4598 and ICG2777)—were sown in three replicated plots. Three densities (25, 33, and 50 plant m^-2^) were tested in plots with row-to-row distance of 33 cm and space between plants in the row spaced at 12, 9, and 6 cm, respectively. This trial was also kept fully irrigated.

## Results

### Comparison of Simulations with Observed and Marksim-Generated Weather Data

**FIGURE 1:**
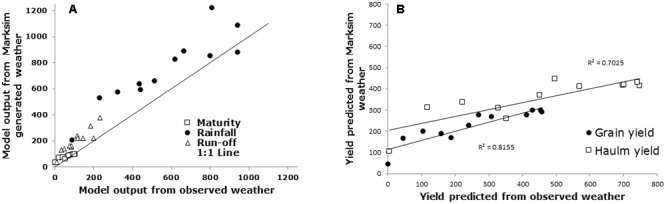
**Comparison of rainfall from observed weather at 12 locations in Mali and Marksim-generated rainfall at these locations** (**A**, closed circles), run-off at these locations (**A**, open triangles), and resulting predictions by the crop model of time to maturity (**A**, open square), grain yield (**B**, closed circles), and haulm yield (**B**, open squares).

On average across the 12 locations in Mali, the average rainfall received during the cropping season was 569 mm, whereas Marksim generated wetter climate (about 170 mm on average) with an average across locations of 743 mm during the cropping season. Yet, Marksim generated rainfall were in agreement with the observed rainfall (*R*^2^ = 0.82; **Figure 1A**) and if Marksim generated weather that was wetter than in the observed locations, this discrepancy was fairly consistent across a gradient of rainfall from less than 200 mm to more than 1000 mm during the season. However, the Marksim weather also led to a higher run-off (199 versus 110 mm on average; **Figure 1A**), indicating that while Marksim rains were higher, more than half of it was lost in run-off, therefore largely diminishing the potential effect of the higher Marksim rainfalls. Using both Marksim generated weather and observed weather, the model was used to predict the duration of the crop (maturity) and showed predictions that were also in close agreement (**Figure 1A**).

The pod yield predicted from the observed weather was on average 257 g m^-2^ across the 12 locations and this was in close agreement with an average grain yield of 224 g m^-2^ simulated from the Marksim weather (RMSE = 99 g m^-2^). The haulm yield followed a reverse trends but this was also in agreement, i.e., 453 g m^-2^ using the observed weather versus 352 g m^-2^ using the Marksim weather (RMSE = 198 g m^-2^). There was also a close correlation between the grain yield simulated from the observed weather and that simulated from the Marksim weather (*R*^2^ = 0.82 for grain yield and *R*^2^ = 0.70 for haulm yield; **Figure 1B**), indicating that the sensitivity of the model to simulate grain and haulm yield in response to increases in rainfall in this gradient of latitude, was equally sensitive in predicting grain yield using the weather data generated by Marksim.

**FIGURE 2:**
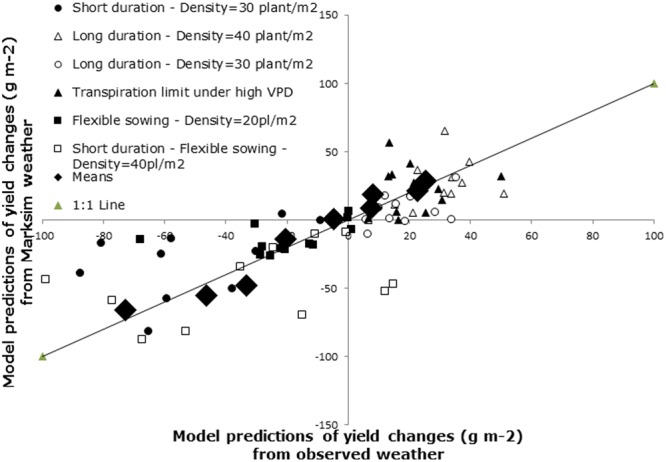
**Model predictions of yield changes from six trait/trait combinations using either observed weather or weather generated by Marksim.** The diamond represents the mean of the model predictions across the available weather (30–50 years for the observed weather and 50 years for Marksim).

The purpose of generating weather data was mostly to be able to run simulations across a wide geographical track without having any bias in the quality and heterogeneity of weather data. As we have seen above, there was a small degree of error in the simulations and our interest was not so much in being able to predict absolute yield values, but rather in being able to predict a change in the grain or haulm yield, either positive or negative, following either a change in rainfall, or an alteration in a genetic or agronomic coefficients of the crop used by the model. **Figure 2** therefore illustrate the changes in grain yield following an alteration in six coefficients or groups of coefficients (combining both genetic and agronomic alterations). Three alterations led to an increase in the grain yield across locations: setting up a limit on transpiration above a VPD response threshold, and sowing at a higher density with long duration cultivars (**Figure 2**). In all cases, these positive increases were predicted both with the output coming from the simulations using observed weather data and with simulations using Marksim weather data. Reversely, three other alterations led to a decrease in grain yield across locations: combinations of lower sowing densities and shorter cultivar durations, in both cases using a flexible sowing window. Similar to the three previous alterations, in all cases, these yield decreases were predicted both with the output coming from the simulations using observed weather data and with simulations using Marksim weather data. These changes were averaged across the 12 locations (closed black symbols) and these averages correlated very closely (*R*^2^ = 0.95—large diamond symbols) between those obtained from simulation with observed weather and those obtained with simulated weather. In addition, the correlation of averages was closely aligned to the 1:1 line, indicating that while absolute simulation values of grain and haulm yield differed somewhat between those generated from observed and simulated weather (see **Figure 1B** for instance), the changes in yield caused by any genetic or agronomic alteration was extremely precisely pinpointed with Marksim weather data (**Figure 2**).

### Baseline Yields under Rainfed Conditions and Yield Reductions Caused by Water Deficit

Simulations were run in two large blocks of West Africa and South East Africa. In West Africa, rainfed grain yield ranged from about 100 g m^-2^ in the most northern fringes (15° latitude) up to about 360 g m^-2^ in the most Southern fringes. Groundnut yield in countries like Senegal, Mali, and Niger were well under 2 tons/ha whereas groundnut productivity in countries like Nigeria or Ivory Coast, located more at the South, were well above 3 tons/ha. Therefore, there was a clear gradient of decreasing grain yield while moving up in latitude (Supplementary Figure 1a). In South East Africa, the block contained countries such as Kenya, Uganda, and Sudan where the rainfall patterns does not fit a window of sowing time for the late part of the year and these should be ignored. In the remaining part of the block, grain yield ranged between 125 g m^-2^ up to about 330 g m^-2^. Different from the case of West Africa, there was no clear latitude or longitude gradient in the grain yield except yield going down while going North in Tanzania (Supplementary Figure 1b).

**FIGURE 3:**
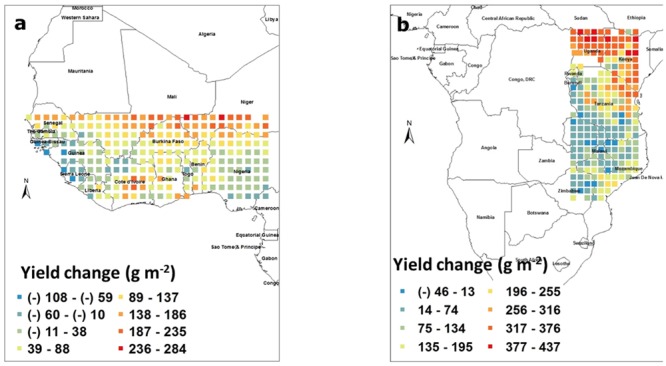
**Groundnut pod yield changes (g m^-2^) caused by water deficit in West Africa (a)** and in South East Africa **(b)**. Each square represents the differences in the means of simulated outputs under rainfed conditions subtracted from those under fully irrigated conditions. These means were those of simulated outputs over 50 years of weather data generated by Marksim. The color coding provides indications of the range of yield changes in the different squares.

To estimate the yield losses caused by water deficit, the model was run to irrigate the crop each time the FTSW fell below 0.35, and yield simulated under rainfed conditions was subtracted from the yield simulated for fully irrigated conditions. Yield losses caused by water deficit in West Africa ranged from -100 to 245 g m^-2^. It is not clear why there was a yield penalty caused by irrigating the crop and this might be caused by the model bringing symbiotic nitrogen fixation to 0 in soil where FTSW is equal to 1 (soil fully saturated), which could have occurred if irrigation event were followed by rainfall events. Another possibility is for a larger leaf area development in fully irrigated plants, which was detrimental toward the end of the season in case of late water deficits. In fact, there is evidence in Virginia groundnut of an increase in vegetative biomass upon increased density not always leading to increased yield because of indeterminacy in pod setting (Cahaner and Ashri, 1974). In any case, even if losses caused by water deficit could be large, these were mostly limited to latitudes above 13° North and as such, countries like Senegal, Mali, and Niger would have most of their groundnut production suffering from severe water deficit. By contrast, a large majority of the pixels in that block were not suffering from water deficit, for example Nigeria (**Figure 3a**). In the South East block, again excluding Kenya, Uganda, and Sudan, the yield penalty caused by water deficit ranged from about -50 to 225 g m^-2^. The negative effect of irrigation could also have been caused by symbiotic nitrogen fixation being inhibited in fully saturated soils. Here also, a large majority of the block showed only a minor yield penalty caused by water deficit, except the Northern part of Tanzania and the South East part of Mozambique where water deficit had a substantial effect on yields with penalty ranging commonly from 1 to 2 tons/ha (**Figure 3b**).

### Simulating the Effect of Increasing the Sowing Density from 20 to 40 Plant m^-2^ on Yield

**FIGURE 4:**
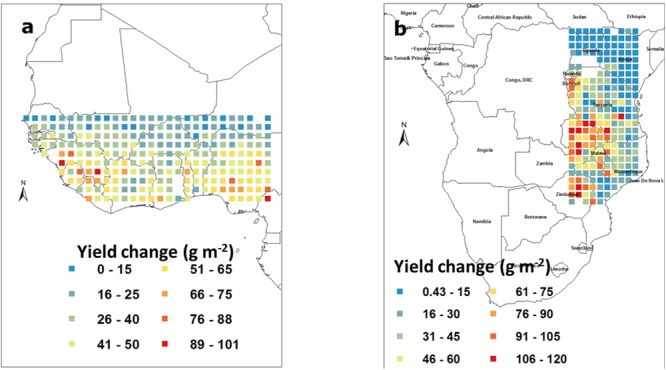
**Groundnut pod yield change (g m^-2^) caused by increasing the sowing density from 20 to 40 plants rrr^2^ in West Africa (a)** and in South East Africa **(b)**. Each square represents the means of simulated outputs under 40 plants nr^2^ subtracted from those under 20 plants nr^2^. These means were those of simulated outputs over 50 years of weather data generated by Marksim. The color coding provides indications of the range of yield changes in the different squares.

Similarly to the above case, model simulations were run with a higher sowing density and the grain yield from the simulation under lower density were subtracted from those under higher density for each pixel. In West Africa, the increase in yield caused by increasing plant density were positive in all cases and ranged between 15 and 100 g m^-2^. These yield increased were below 40 g m^-2^ in countries like Senegal, Mali, and Niger. By contrast, countries like Nigeria or Ivory Coast showed a major yield increase from increasing the sowing density (**Figure 4a**). Similar observations were made in the South East block where yield increase were positive in the entire block and ranged from 15 to 120 g m^-2^. Here also, there was a clear East/West gradient in the effect of increasing sowing density, with the Eastern parts of Tanzania and Mozambique benefiting little from an increase in density, whereas countries like Zambia, Zimbabwe, and the Western part of Tanzania would benefit a lot from increase planting density (**Figure 4b**).

For farmers having to decide whether to adopt a new management practice, it is important to be able to provide guidance on the percentage of time this change would bring a positive outcome. Indeed, no single intervention can guaranty success in all years. Therefore the probability that a yield increase would occur from a doubling of the planting density was calculated. In the West Africa region, yield increase would occur in at least 80–90% of the cases in all regions. Therefore in West Africa farmer growing groundnut in non-water-limited conditions with fertilizer would benefit from higher sowing densities with a high probability of success. These percentages, although high, were somewhat lower in the highest latitudes and lower rainfall environments (Supplementary Figure 2a). The results were very similar in the South East Africa block, where increasing density would be beneficial in at least 80–90% of the environments. Only the Eastern parts of Tanzania and Mozambique would see slightly lower probabilities (78–80%; Supplementary Figure 2b).

### Testing the Robustness of the Simulations of Density Effects Experimentally

**FIGURE 5:**
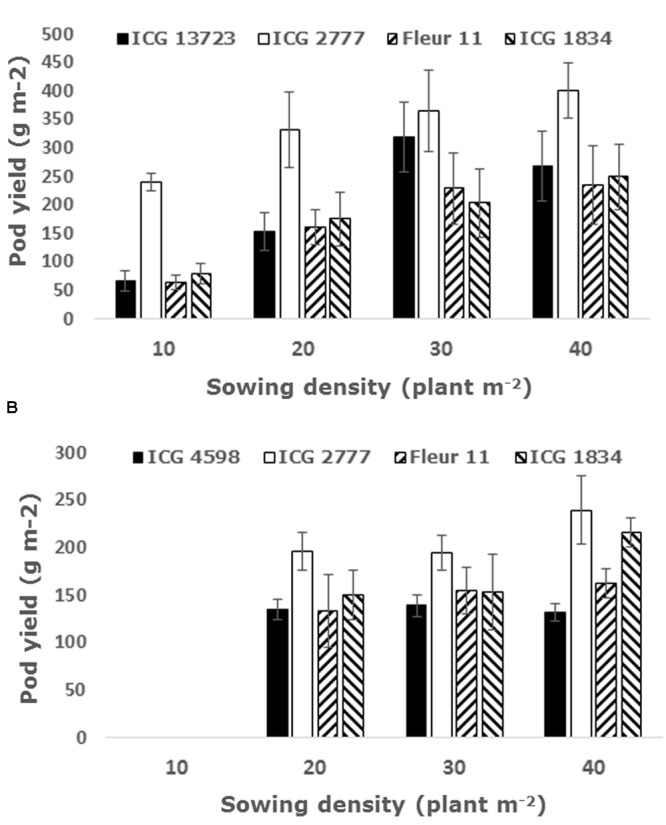
**Groundnut pod yield in two density experiments carried out in Niger during the rainy season with four sowing densities (A)** and India late in the rainy season with three sowing densities **(B)**, in both cases with two Virginia cultivars (black and white bars) and two Spanish genotypes (striped bars). Data are means (±SE) of three replicated plots in each genotype-by-density combination.

So far the most promising avenue for increasing groundnut grain yield from the model output came from increasing the sowing density and this was tested experimentally in two experiments in Niger and India. In Niger, there was a clear trend of increasing pod yield with increasing densities, although the effect differed between genotypes. The two Virginia genotypes reached the highest pod yield at 20 and 30 plant m^-2^, whereas the two Spanish cultivars reached the highest yield at 30 plant m^-2^ (**Figure 5A**). In India, the lowest density was not used and the Virginia genotypes showed no significant differences in the pod yield across densities, while there was an increase in the pod yield up to 40 plant m^-2^ in one of the Spanish cultivars (**Figure 5B**). Old density trial data were also consulted (Waliyar, personal communication), and confirmed the positive effect of increasing sowing density on yield, also showing a different magnitude of response across cultivars.

### Re-Assessing Yield Reductions Caused by Water Deficit under Optimum Sowing Densities

**FIGURE 6:**
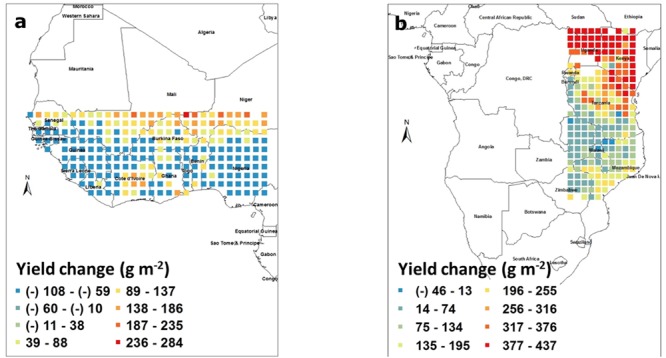
**Re-assessment of the effect of water deficit on pod yield of groundnut (g m^-2^) caused by water deficit in West Africa (a)** and in South East Africa **(b)**, where a density of 40 plant m^-2^ was used. Each square represents the differences in the means of simulated outputs under rainfed conditions subtracted from those under fully irrigated conditions. These means were those of simulated outputs over 50 years of weather data generated by Marksim. The color coding provides indications of the range of yield changes in the different squares.

Increasing the sowing density was the factor leading to the highest yield changes among those that were tested. Despite the fact that water deficit had only a mild effect on groundnut yield, except in specific regions, it was important to confirm whether water could have become limiting under those increased densities. In West Africa, at these higher densities, the yield losses causes by water deficit ranged from -108 to 284 g m^-2^. Here also there was a yield penalty caused by irrigating the crop similar to **Figure 3**. Yield losses caused by water deficit were mostly restricted to latitudes above 13° North (**Figure 6a**) and the delimitation of the regions affected by water deficit was much clearer than under lower densities as in **Figure 3a**. As such, only the North part of Senegal, Mali, this time Burkina Faso, and Niger would have most of their groundnut production suffering from severe water deficit. By contrast, a large majority of the remaining pixels in that block were not suffering from water deficit, for example, Nigeria (**Figure 6a**). In the South East block, again excluding Kenya, Uganda, and Sudan, the yield penalty caused by water deficit under these increased densities ranged from about -50 to 225 g m^-2^. There was no major change in the map as compared to lower density (**Figure 3b**). Here also, a large majority of the block showed only a minor yield penalty caused by water deficit, except the Northern part of Tanzania and the South East part of Mozambique where water deficit had a substantial effect on yields with penalty ranging commonly from 1 to 2 tons/ha (**Figure 6b**).

### Simulating the Effect of Shortening the Duration of the Crop Cycle

**FIGURE 7:**
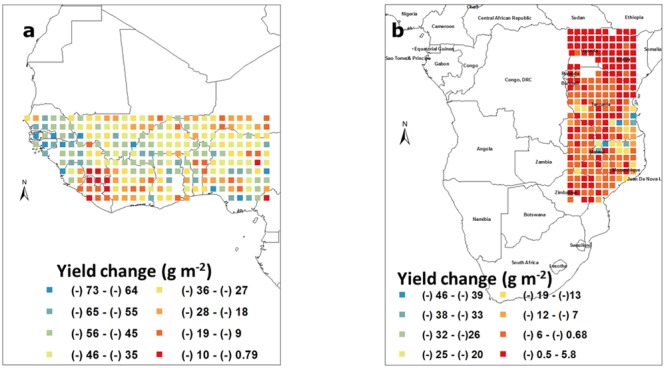
**Groundnut pod yield changes (g m^-2^) caused by shortening the crop duration by 10 biological days in West Africa (a)** and in South East Africa **(b)**. Each square represents the differences in the means of simulated outputs under a regular crop duration subtracted from those of a shortened crop cycle. These means were those of simulated outputs over 50 years of weather data generated by Marksim. The color coding provides indications of the range of yield changes in the different squares.

In West Africa, reducing the duration of the crop cycle by 10 biological days led to yield losses ranging between 10 and 70 g m^-2^. There was in this case no clear gradient in the yield reduction, except some countries like Senegal suffering the most from growing early duration lines (**Figure 7a**). In the South East Africa block, there were virtually no major change from growing early duration cultivars, except in the Eastern part of Tanzania where mild losses of around 25–40 g m^-2^ could be detected (**Figure 7b**).

## Discussion

### Yield Potential

The model provided a very useful assessment of the yield potential for peanut in two large geographical blocks of SSA where groundnut is commonly cultivated. It gave a clear view of where groundnut could be of high economical returns, like in Nigeria and then calls for testing these predictions on the ground as was done for the sowing density aspects. These predicted rainfed yield were far from the average yield in the region (less than 1 ton ha^-1^), indicating fairly high yield gaps. The cause for these yield gaps are not clear although low soil fertility, especially low soil phosphorus, and foliar diseases are well-known factors decreasing yields in the region. A 20-year-old study (Waliyar, personal communication) in the region showed a beneficial effect of treating groundnut for foliar diseases on pod yield, and the fertilizer application in Africa is less than 20 kg ha^-1^. As was studied here, a major cause for this yield gap might be in the improper seed rate used, leading to planting density that is sub-optimal. Although this was not the purpose of the paper, using this crop model to undertake an exhaustive yield gap analysis in the Sub-Saharan region would be the next step to the current baseline study.

### Impact of Drought

It is a fact that many of the semi-arid tropical regions of Africa receive limited rainfall but part of these regions receive rainfall higher than the water required for a groundnut crop to reach its yield potential based on recent lysimetric assessments (Halilou et al., 2015). Nevertheless, water deficit, or so-called “drought” is considered as the main abiotic stress affecting groundnut pod yield and is the object of a lot of research. The purpose of the study was to properly delimit the areas where water is indeed limiting and requires research attention and investment. Again, the model turned out to be useful to offer a geographical representation of the areas, or countries, where the groundnut crop indeed suffered water deficits. As such, the model predicted that in most pixels of the two geographical blocks, water deficit was only a limited problem. It was an issue only at latitudes above 13° North in WCA (above 12° under an optimal sowing density) where groundnut cultivation becomes limited, and only in the most Eastern fringes of Tanzania and Mozambique. These results were made even clearer under higher sowing density conditions (**Figure 6a**), where the boundaries delimiting water stress areas fell down to 12° latitude, and therefore making of North Senegal, Burkina Faso, Mali, and Niger countries very affected by water stress for groundnut production. Therefore, these results are extremely powerful to orient breeding targets and make these more focused to specific regions for specific limitation.

It could be argued that Marksim generated wetter weather (about 170 mm more on average) that could have influenced the response in water limited environments. However, the run-off of Marksim generated weather was also much higher, i.e., about 90 mm more, than the observed weather. In other words, more than half of the extra rain from Marksim was lost in run-off. Therefore, while the rainfall value differences between Marksim and observed weather could not be ignored, the high run-off in Marksim weather likely diminished the possible effects of higher rainfall on stress scenarios and boundaries. This would also explain why the simulations from both types of weather agreed quite closely and if simulations for rainfall below 300 mm (observed rainfall) were higher with Marksim than with observed weather (**Figure 1B**), the simulations with Marksim were still able to detect the effect of a water deficit at these latitudes. In addition, as explained earlier, our objective was about tracking possible changes occurring upon changes in either the agronomy or the genetics of the crops, and there Marksim appeared to be well able to detect the effect of these changes on yield equally well than with observed weather (**Figure 2**).

The large differences between the predicted rainfed yield and the average regional yield also imply that the limitation to peanut production in these regions is mostly other than drought. Water availability over there would likely be more than sufficient to support an excellent groundnut crop, provided the other factors such as soil fertility and foliar diseases are prevented, and as was shown, provided an adequate sowing density is used (see below). No attempt was made to predict what would happen in future climate as this would be a study on its own and the focus was on possible improvements to be made “today.” Such exercise would also be made difficult by the lack of a clear understanding of the effect of climate change on rainfall patterns and intensity. We could only anticipate the effects would be an interplay of the increased temperature (very likely increase in VPD with effects on the plant water balance and hastening of the crop cycle) against alleviation effects from an increased CO_2_ concentration. The effect of soil type was also not tested, the objective being to have an overall representation of water stress effect in a crop that grows on received rainfall and where stored moisture plays a lesser role. Further study could explore these effects at a much lower scale (country or sub-region) to take into account soil type and fine tune water stress boundaries.

### Increasing Sowing Density Causes a Major Yield Increases

The other main result from this study was in showing that doubling the sowing density from 20 to 40 plant m^-2^ (from about 60–120 kg seed ha^-1^) would bring about major yield benefits. These modeling results were confirmed experimentally in Niger. They also agree with similar results from trials in the WCA region in the mid-nineties (Waliyar, personal communication), where a combination of increasing the sowing density with foliar disease sprays showed both a positive effect of increasing the sowing density, especially when the crop was also treated for foliar diseases. One reason for the slower agronomic changes in SSA could come from the higher proportion of Virginia runner types being cultivated in SSA and adapted to lower sowing density than Spanish types, as shown in earlier studies (Cahaner and Ashri, 1974; Tewolde et al., 2002). These previous studies also showed a degree of genotype-by-density interaction, and so did our experimental data where the response to increasing density was not shown in all entries. Another reason is the cost of seeds. Peanut is one such crop that requires large amount of seeds and our interpretation is that farmers weigh out the risk of investing in larger seed quantities versus the expected return. As such, we may expect that unless yield increases are large, say, higher than half ton, there would likely be limited interest.

These results bring an interesting discussion on what should be the breeding and agronomic target for the SSA region, where Spanish types with an erected phenotype are being predominantly cultivated. First, a seed rate increase implies an additional investment that farmers may not be able to bear, or a risk to which they are averse. Second, groundnut production often re-uses the ridges of the previous tobacco or cotton production, which are 60 cm apart or so. Therefore, increasing the density implies both cost and possibly additional labor for re-ridging the field. Extension interventions would then be needed to alleviate these trade-offs, for instance providing credit for seeds, or innovations to allow a higher sowing density on the current ridging management (like sowing on both sides of the ridge). This analysis suggests that if indeed the seed and labor trade-offs are sufficient to hamper adoption of new agronomic practices, the opportunity for improving pod yield would come from growing a plant type able to cover the ground quickly to limit soil evaporation and optimize radiation interception. Our interpretation is that under such conditions, current genetic improvement efforts, largely focused on Spanish types, should probably recast their focus on runner types that offer a phenotype adapted to low densities. If high potential areas were keen on increasing the density using Spanish types, these would then probably need additional genetic efforts, not so much for improving tolerance to water deficit, but rather to address the reasons for the large yield gaps, i.e., foliar diseases and low soil fertility.

## Author Contributions

VV designed the study and wrote the paper, OH and HH ran the field experiments, PS-T provided the weather data, TS and AS provided modeling support and help in the design of the experiment. All authors read and approved the paper

## Conflict of Interest Statement

The authors declare that the research was conducted in the absence of any commercial or financial relationships that could be construed as a potential conflict of interest. The reviewer SA declared a past collaboration with one of the authors PS-T to the handling Editor, who ensured that the process met the standards of a fair and objective review.
