# Supplementary figures and images for: Mapping Water Stress Incidence and Intensity, Optimal Plant Populations, and Cultivar Duration for African Groundnut Productivity Enhancement
Source: Front Plant Sci. 2017 Mar 29;8:432. doi: 10.3389/fpls.2017.00432 (PMC5370244; doi:10.3389/fpls.2017.00432)

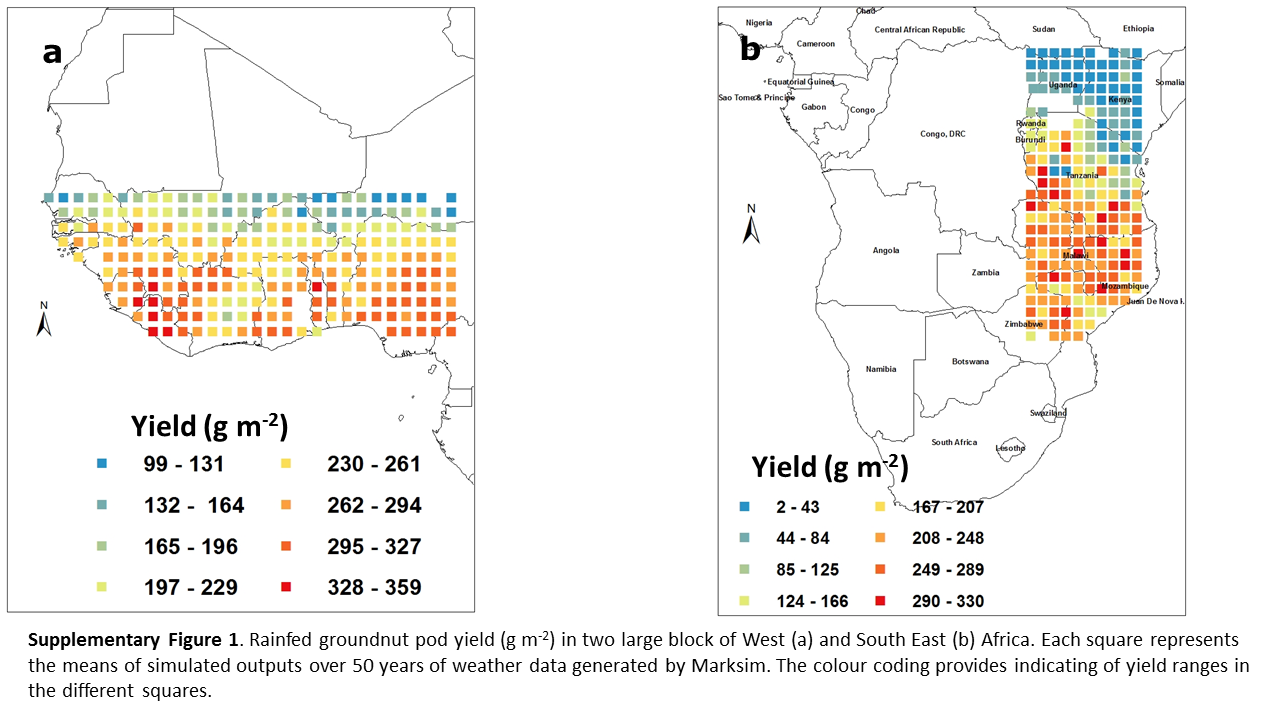

Supplement: Supplementary file 1 [file Image_1.TIF]

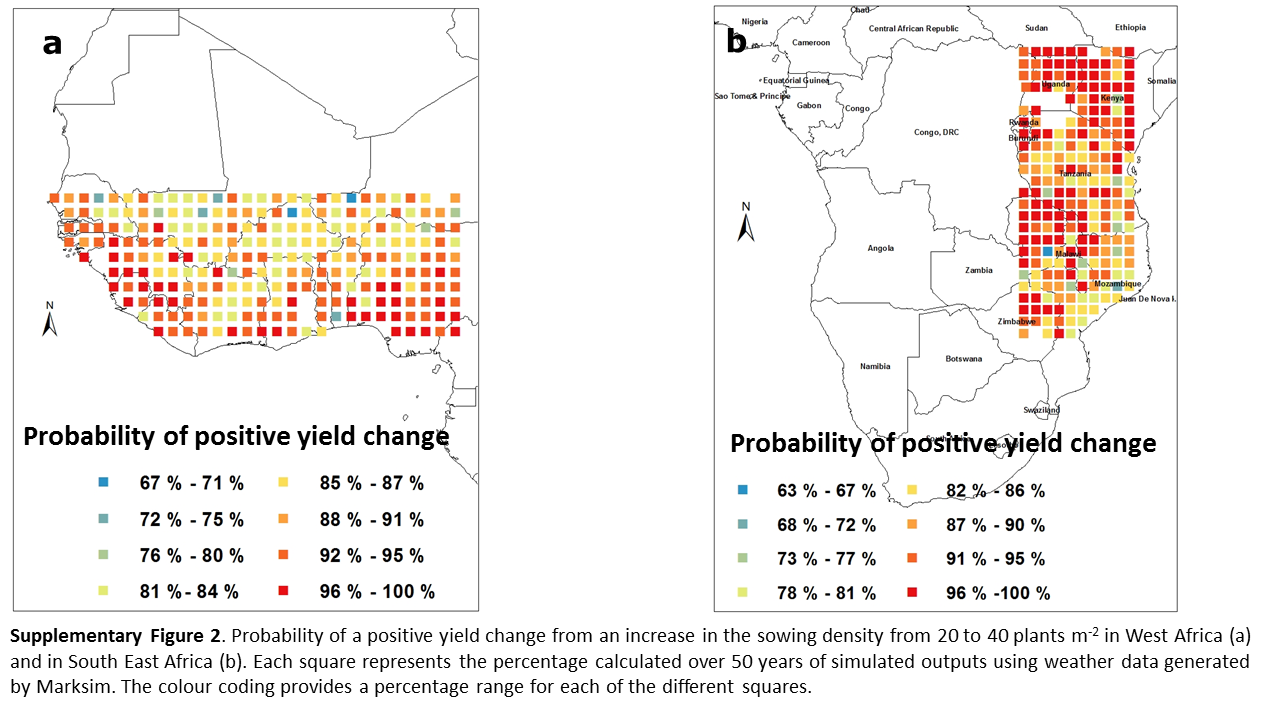

Supplement: Supplementary file 2 [file Image_2.TIF]
